# Identification and Validation of the Diagnostic Characteristic Genes of Ovarian Cancer by Bioinformatics and Machine Learning

**DOI:** 10.3389/fgene.2022.858466

**Published:** 2022-06-01

**Authors:** Jinya Liu, Leping Liu, Paul Akwasi Antwi, Yanwei Luo, Fang Liang

**Affiliations:** ^1^ Department of Plastic Surgery, The Third Xiangya Hospital of Central South University, Changsha, China; ^2^ Department of Blood Transfusion, The Third Xiangya Hospital of Central South University, Changsha, China; ^3^ Department of Hematology and Critical Care Medicine, The Third Xiangya Hospital, Central South University, Changsha, China

**Keywords:** ovarian cancer, machine learning, immune cell infiltration, LASSO, SVM, diagnosis

## Abstract

**Background:** Ovarian cancer (OC) has a high mortality rate and poses a severe threat to women’s health. However, abnormal gene expression underlying the tumorigenesis of OC has not been fully understood. This study aims to identify diagnostic characteristic genes involved in OC by bioinformatics and machine learning.

**Methods:** We utilized five datasets retrieved from the Gene Expression Omnibus (GEO) database, The Cancer Genome Atlas (TCGA) database, and the Genotype-Tissue Expression (GTEx) Project database. GSE12470 and GSE18520 were combined as the training set, and GSE27651 was used as the validation set A. Also, we combined the TCGA database and GTEx database as validation set B. First, in the training set, differentially expressed genes (DEGs) between OC and non-ovarian cancer tissues (nOC) were identified. Next, Gene Ontology (GO), Kyoto Encyclopedia of Genes and Genomes (KEGG), Disease Ontology (DO) enrichment analysis, and Gene Set Enrichment Analysis (GSEA) were performed for functional enrichment analysis of these DEGs. Then, two machine learning algorithms, Least Absolute Shrinkage and Selector Operation (LASSO) and Support Vector Machine-Recursive Feature Elimination (SVM-RFE), were used to get the diagnostic genes. Subsequently, the obtained diagnostic-related DEGs were validated in the validation sets. Then, we used the computational approach (CIBERSORT) to analyze the association between immune cell infiltration and DEGs. Finally, we analyzed the prognostic role of several genes on the KM-plotter website and used the human protein atlas (HPA) online database to analyze the expression of these genes at the protein level.

**Results:** 590 DEGs were identified, including 276 upregulated and 314 downregulated DEGs.The Enrichment analysis results indicated the DEGs were mainly involved in the nuclear division, cell cycle, and IL−17 signaling pathway. Besides, DEGs were also closely related to immune cell infiltration. Finally, we found that BUB1, FOLR1, and PSAT1 have prognostic roles and the protein-level expression of these six genes SFPR1, PSAT1, PDE8B, INAVA and TMEM139 in OC tissue and nOC tissue was consistent with our analysis.

**Conclusions:** We screened nine diagnostic characteristic genes of OC, including SFRP1, PSAT1, BUB1B, FOLR1, ABCB1, PDE8B, INAVA, BUB1, TMEM139. Combining these genes may be useful for OC diagnosis and evaluating immune cell infiltration.

## Introduction

Ovarian cancer (OC) is the eighth leading cause of cancer death and the seventh-most frequently diagnosed among females worldwide (Torre et al., 2015; Coburn et al., 2017). Despite other cancers, such as cervical cancer, having higher rates of incidence, ovarian cancer mortality rates continue to be high (Siegel et al., 2020). As early-stage tumors symptoms are typically asymptomatic (Chan et al., 2022; Zhang & Hu, 2022), more than 70% of OC cases are diagnosed at an advanced stage (Chern & Curtin, 2016; Sung et al., 2021). In most countries, the 5-year survival rate of OC is usually lower than 40% (Vaughan et al., 2011). The high morbidity and mortality rates make early screening and diagnosis of ovarian cancer even more important. Cancer diagnostic genes are closely related to tumor diagnosis and prognostic survival. Ovarian cancer candidate diagnostic genes and tumor microenvironment immune genes are still unclear, so this study chooses the machine learning approach to predict ovarian cancer diagnostic genes to provide some help for the early diagnosis of ovarian cancer.

The occurrence and development of ovarian cancer are affected by the tumor microenvironment (Jiang et al., 2020; Zhang et al., 2021). Among the tumor microenvironment, immune cells are the key factors of tumor progression. At the same time, immunotherapy is a promising tumor-killing method (Zhu et al., 2021). The degree of infiltration of immune cells can reflect the response of ovarian cancer cells to immunotherapy, as well as different prognoses (Goode et al., 2017). However, despite the development of immunotherapy for ovarian cancer, the results have not been satisfactory. Immune cell infiltration and distribution are highly heterogeneous and complex, and the search for factors driving immune infiltration or key biomarkers is crucial to reveal this heterogeneity (Odunsi, 2017; Cai et al., 2021; Faust et al., 2022). Therefore, studying the infiltration state of immune cells and discovering new immune-related characteristic genes is essential for the treatment of ovarian cancer.

In recent years, machine learning has been applied to various fields of biomedicine. Compared with most traditional statistical methods, the advantage of machine learning is that it can identify potential rules through massive data learning (Zhao et al., 2020). With the development of high-throughput sequencing technology, the efficiency of gene sequencing has improved exponentially, and thus machine learning can also be well applied to identify cancer characteristic genes. Machine learning algorithms have been applied to identify cancer prognostic characteristic genes and tumor classification. Cangelosi et al. (2014) used the Logic Learning Machine algorithm to determine the prognostic characteristic genes associated with neuroblastoma (Cangelosi et al., 2014). Liu et al. (2021) used a variety of machine learning algorithms to identify a ferroptosis-related lncRNA signature for lung adenocarcinoma and three pyroptosis-related molecular subtypes of lung adenocarcinoma (Liu et al., 2021; Lu et al., 2021). All these findings show the great potential of machine learning in oncology research. It can learn high-dimensional gene expression data to perform specific classification tasks. However, there is little research on machine learning in identifying characteristic genes related to cancer diagnosis, hence, a further in-depth study is required.

In this study, based on three cohorts from the GEO dataset, we analyzed differential genes between ovarian cancer tissues and non-tumor tissues, and explored the biological functions and pathways involved in these differential genes. Two different machine learning algorithms were also used to identify key ovarian cancer diagnostic characteristic genes. Finally, we performed immune cell analysis and association analysis of these key genes and immune cells.

## Materials and Methods

### Datasets

The NCBI-GEO database, TCGA (http://portal.gdc.cancer.gov/) database, and GTEx database are free and public databases containing gene profiles. We retrieved three microarray datasets (GSE12470, GSE18520, and GSE27651) were from the GEO database (https://www.ncbi.nlm.nih.gov/gds/). Microarray data of GSE12470, GSE18520, and GSE27651 were all on account of GPL570 Platforms [(HG-U133_Plus_2) Affyme-trix Human Genome U133 Plus 2.0 Array]. 43 serous ovarian cancer samples and 10 normal peritoneum samples were included in GSE12470. 53 advanced stage, high-grade primary tumor specimens and 10 normal ovarian surface epithelium brushings were contained by GSE18520. GSE2765149 included 43 ovarian cancer (8 serous borderline ovarian tumors, 13 low-grade serous ovarian carcinomas, and 22 high-grade serous ovarian carcinomas) and 6 human ovarian surface epithelia. Then, we defined the ovarian cancer sample as OC, and the non-ovarian cancer tissues sample as nOC. Additionally, we converted the probe matrix into a genes matrix based on the annotation information using the “perl” language. Then, we combined the GSE12470 and GSE18520 cohorts to constitute a training set. Besides, “sva” and “limma” packages in R language were used to do batch correction and to find out the different genes between the OC group and nOC group. Finally, the GSE27651 cohort was used as the validation set A for subsequent validation. Meanwhile, we merged 379 ovarian cancer samples from TCGA and 88 normal ovarian samples from GTEx, as well as batch correction. The merged data set was used as validation set B for subsequent validation. Not only the number of samples can be increased, but also the accuracy of diagnostic genes can be rechecked.

### Differential Expression

According to the data obtained from the GEO database, DEGs screening was performed between the OC group and the nOC group in the training set by the “limma” package. DEGs were found by filtering according to |logFC| > 2 and adj *p* value < 0.01. If logFC >2, it indicated that this gene was upregulated in the OC group. If logFC <−2, it indicated that this gene was down-regulated in the OC group. Moreover, we visualized the analysis results using a volcano plot and a heat map.

### Functional Enrichment Analysis

GO functional analysis, KEGG pathway analysis, DO, and GSEA enrichment analyses were carried out to predict the potential functions of the DEGs by using the “clusterProfiler” “enrichplot” “org.Hs.eg.db” and “ggplot2” in R package. This helps to conclude whether the genes are significantly concentrated in a particular pathway, a particular cytological locus, or a particular class of diseases. These functional enrichment analyses with *p*-value < 0.05 were considered statistically significant.

### Machine Learning

In our research, we needed to create a more accurate prediction system. So, we used two machine learning algorithms, LASSO, and SVM-RFE, to perform feature selection to screen diagnostic markers for OC. LASSO regression is equivalent to ridge regression using 1-parity to address the problem of high-dimensional data sparsity. The analysis is performed in R using functions from the glmnet package, with a model using a binomial model (family = “binomial”) and a loss function using binomial deviance (type.measure = “deviance”). SVM-RFE containing recursive feature elimination recursively removes ground influence factors for better genetic screening. This method is implemented using the cfe function in the “caret” package, and the fitted prediction function is cross-validated. Taking the intersecting genes as the diagnostic genes of the disease, we tested the diagnostic genes in the validation group. If the *p*-value < 0.05, it would mean that these genes differed from the training set and validation set. Furthermore, receiver operating characteristic curve (ROC) was used to observe the accuracy of the disease diagnostic genes.

### Immune Analysis

We used CIBERSORT in R language to analyze the differences in the infiltration of 22 immune cells between the nOC and OC groups. After that, we used diagnostic candidate genes to obtain the relationship between each diagnostic gene and immune cell infiltration. Furthermore, we plotted correlation graphs in the form of scattered points, violin, and lollipop to visualize the data.

### Prognostic Analysis and Protein Expression Analysis

We searched for these diagnostic candidate genes on the KM-plotter (https://kmplot.com/). First, set the tumor type to “ovarian cancer,” then enter the gene name in the search box, and select “midian” in the “Split patients by” column. Finally, select the “user selected probe set” option in the “Probe set options” column.

To further validate our results, we searched the HPA online database (www.proteinatlas.org/) for the protein-level expression of these nine diagnostic candidate genes in ovarian cancer tissues and normal tissues.

## Results

### Screening of Predictive Genes

**FIGURE 1:**
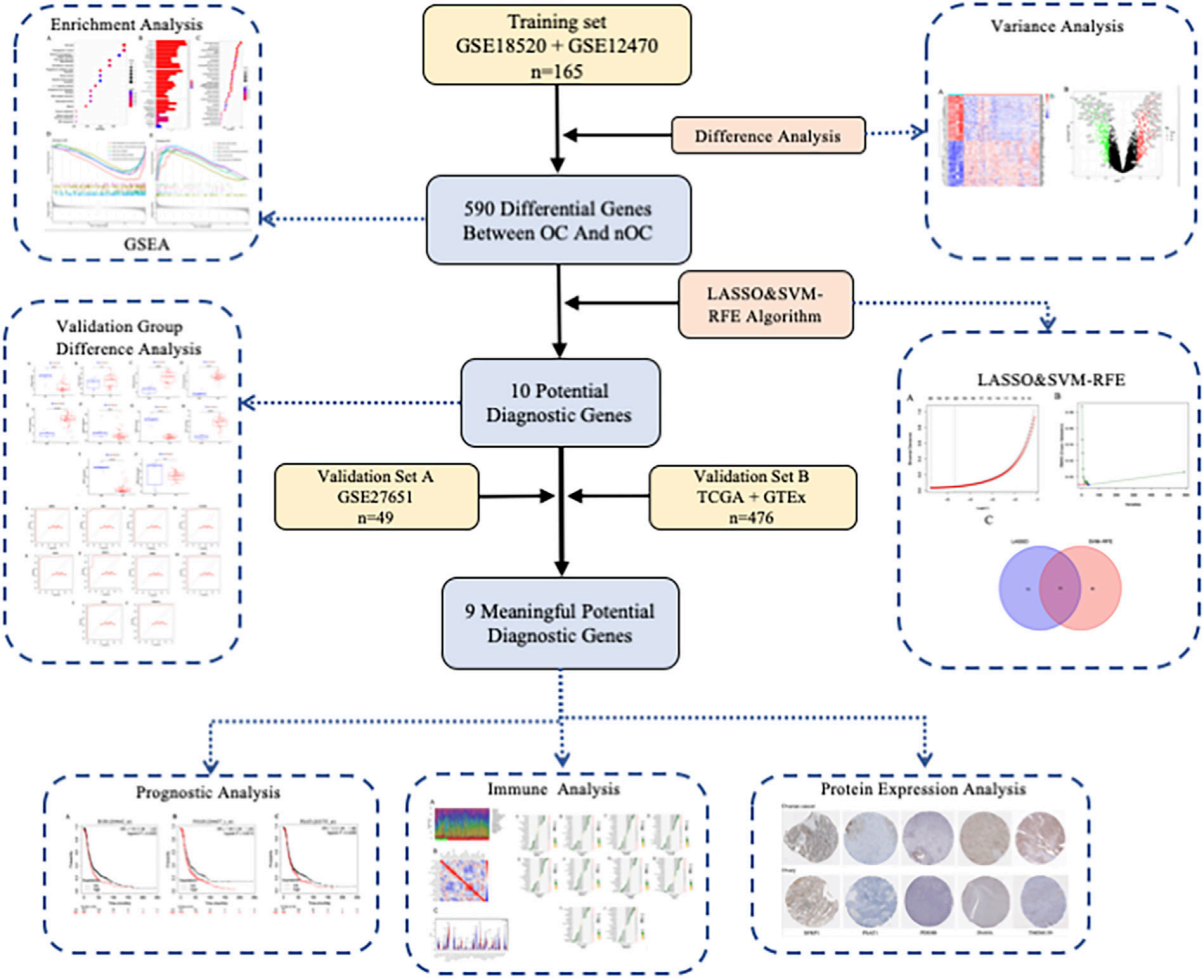
Article framework and workflow.

**FIGURE 2:**
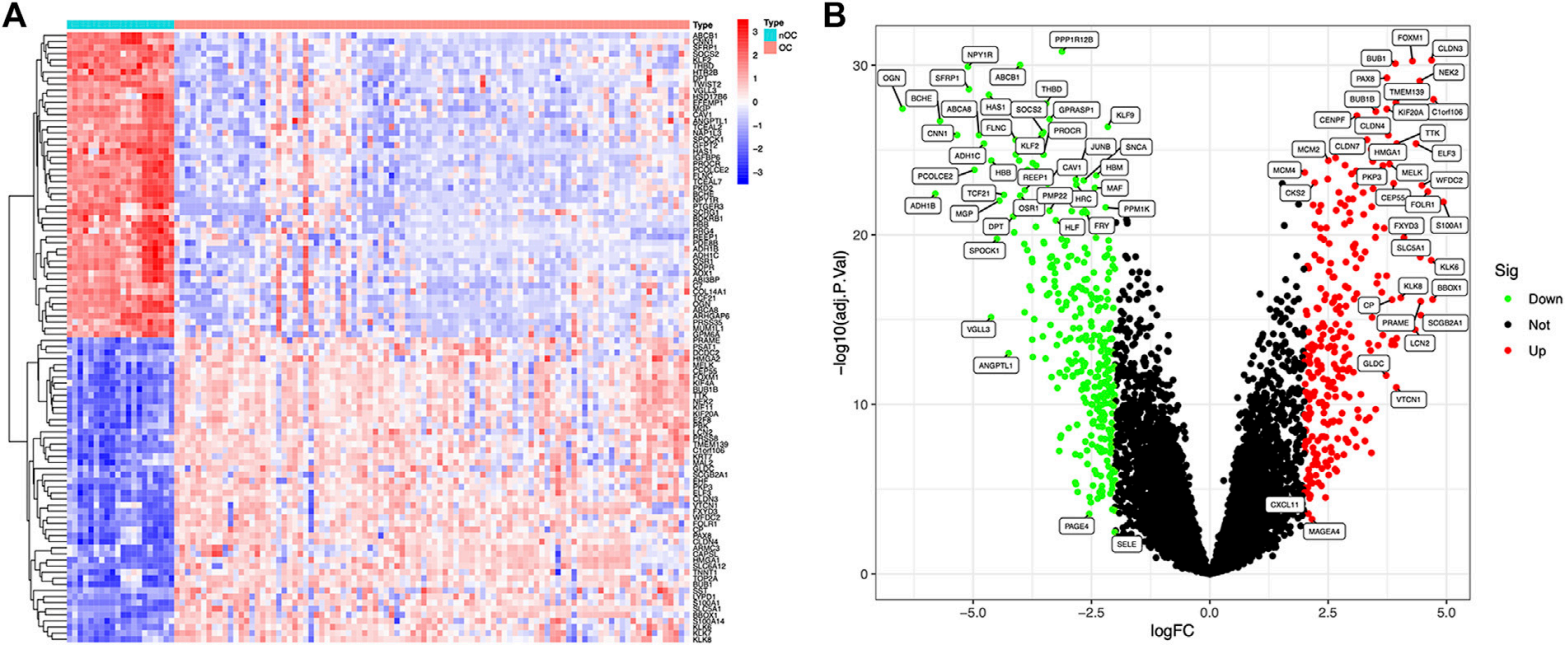
Differentially expressed genes between OC and nOC in the training set. **(A)** The heat map of differentially expressed genes, highly expressed genes were red, and lowly expressed genes were blue. **(B)** The volcano map of differentially expressed genes. Fold changes >2 were indicated by red (upregulation) or green (downregulation).

Article framework and workflow have been systematically described in (Figure 1). 12,881 genes were obtained in OC group and nOC group, and the filtering condition was set to |log2FC| > 2 and *p*-value < 0.05 to obtain 590 DEGs. 50 genes with the most significant upregulation and downregulation were selected. Besides, the heat map and volcano plot were plotted for visualization (Figures 2A,B).

### Enrichment Analysis

**FIGURE 3:**
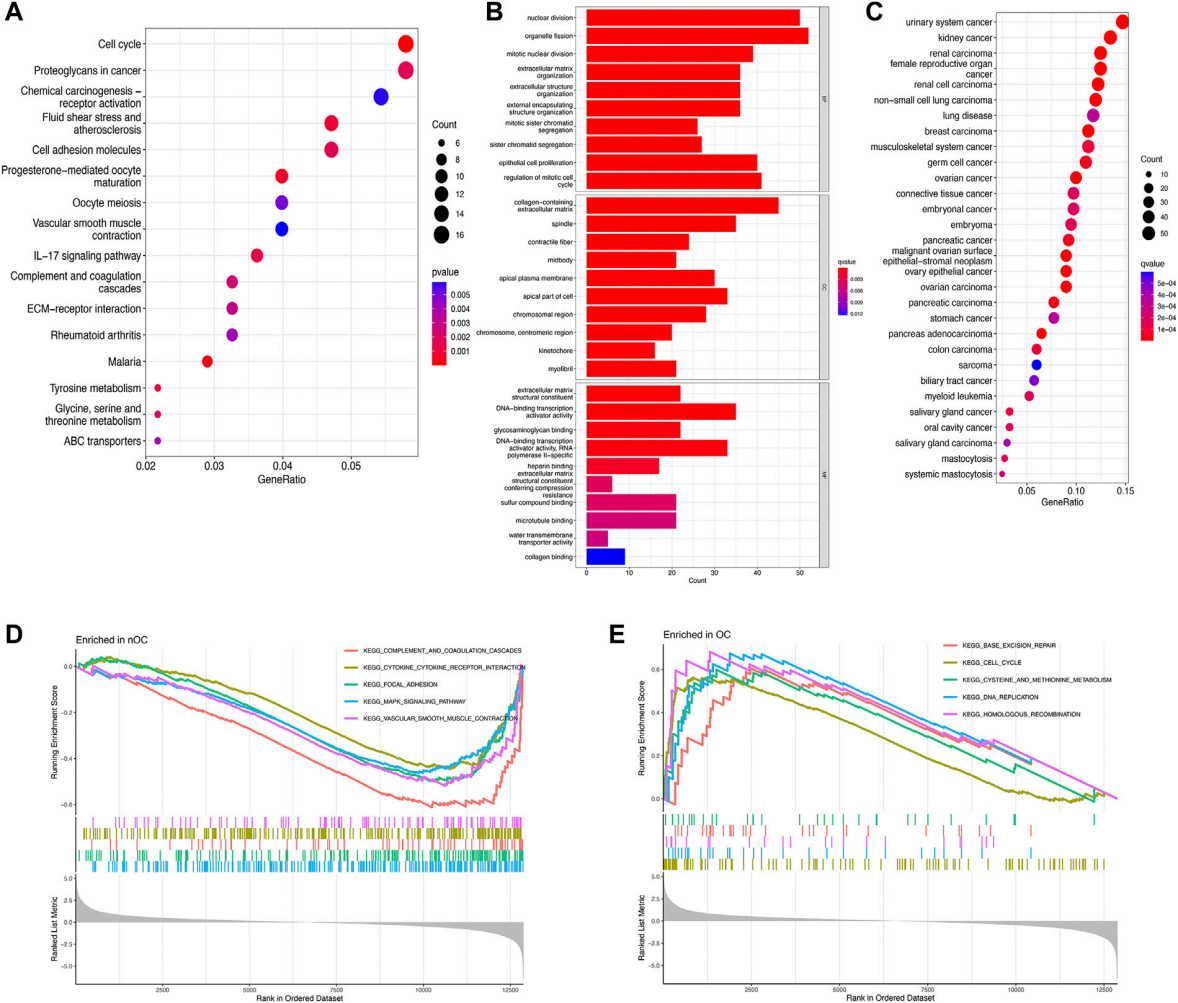
Enrichment analysis of differentially expressed genes. KEGG pathway analysis **(A)**, GO function analysis **(B)**, Disease analysis **(C)**, and GSEA analysis **(D,E)** show the active functions or pathways in the OC and nOC groups, respectively.

To further investigate potential gene functions and signaling pathways between OC and nOC, we extracted 590 DEGs in the training set. Then, GO enrichment analysis, KEGG pathway analysis, GSEA, and DO enrichment analyses were performed based on these DEGs and biological processes with significant enrichment. For KEGG enrichment analysis (*p*-value < 0.05), 16 results with the most significant enrichment of KEGG were selected for bubble visualization. Besides, the signaling pathways with strong association were cell cycle, proteoglycan, progesterone-mediated oocyte maturation, fluid shear stress and atherosclerosis, cell adhesion molecules, and IL-17 (Figure 3A). GO enrichment analysis was performed to determine the enrichment of the functions (adjusted *p*-value < 0.05). The graphs were separated according to biological processes (BP), cell component (CC), and molecular function (MF). The top 10 genes in each of the three groups were selected for visual enrichment analysis (Figure 3B). BP, DEGs were enriched in the nuclear division, cell cycle, extracellular matrix, epithelial growth-related. CC, DEGs were enriched in the collagen deposition, chromosome-related functions. MF, DEGs were significantly enriched in the DNA-binding transcriptional activator activity, RNA polymerase specificity. DO enrichment analysis (adjusted *p*-value < 0.05) obtained the top 30 most significantly enriched diseases plotted as a bubble, with a significant association with urological cancers, female reproductive cancers, and non-small cell lung cancer (Figure 3C). GSEA enrichment analysis was performed to observe the active functional pathways in the OC and nOC groups (adjusted *p*-value < 0.05). The five most active pathways were selected according to the OC and nOC groups, and were plotted separately. The top five active pathways in nOC were complement and coagulation cascade, cytokine and cytokine-receptor-interaction, focal adhesion, MAPK signaling pathway, and vascular smooth muscle contraction (Figure 3D). However, the top five active pathways in OC group were base excision repair, cell cycle, cysteine and methionine metabolism, DNA replication, and homologous recombination pathway (Figure 3D), which were totally different from nOC group.

### Validation Group Difference Analysis

**FIGURE 4:**
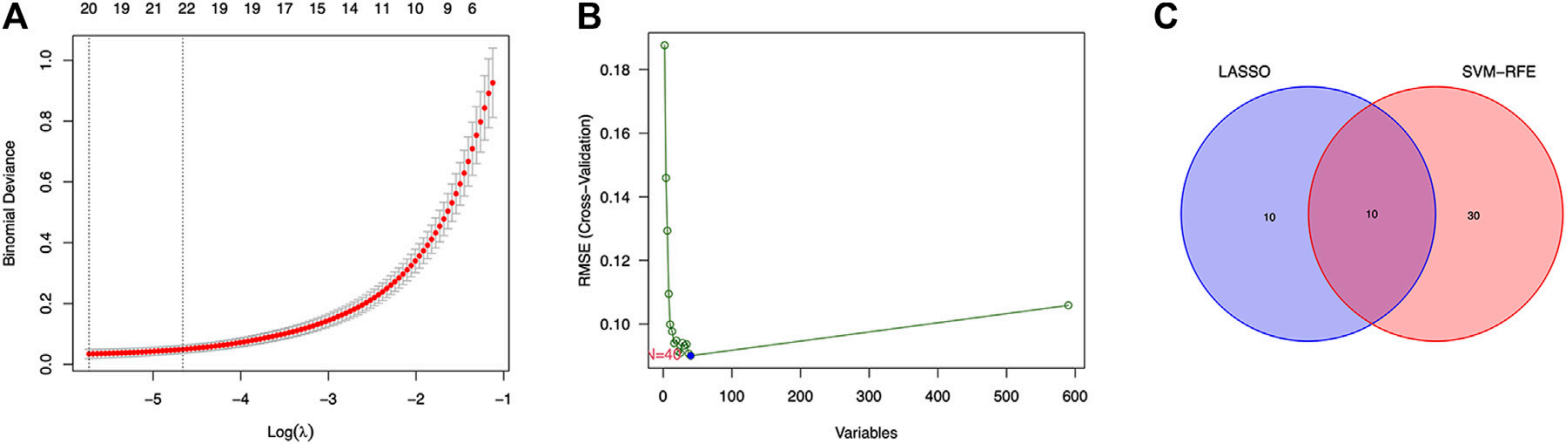
LASSO and SVM-RFE screen characteristic genes. **(A)** LASSO regression screens disease characteristic genes, the abscissa is logλ, and the ordinate is the cross-validation error. When 20 genes are selected, the cross-validation error is the smallest. **(B)** SVM-RFE screens characteristic genes. The abscissa represents the change in the number of genes, and the ordinate represents the cross-validation error. When *n* = 40, the cross-validation error is the smallest. **(C)** Venn diagram shows the intersection of genes found by two machine learning methods.

**FIGURE 5:**
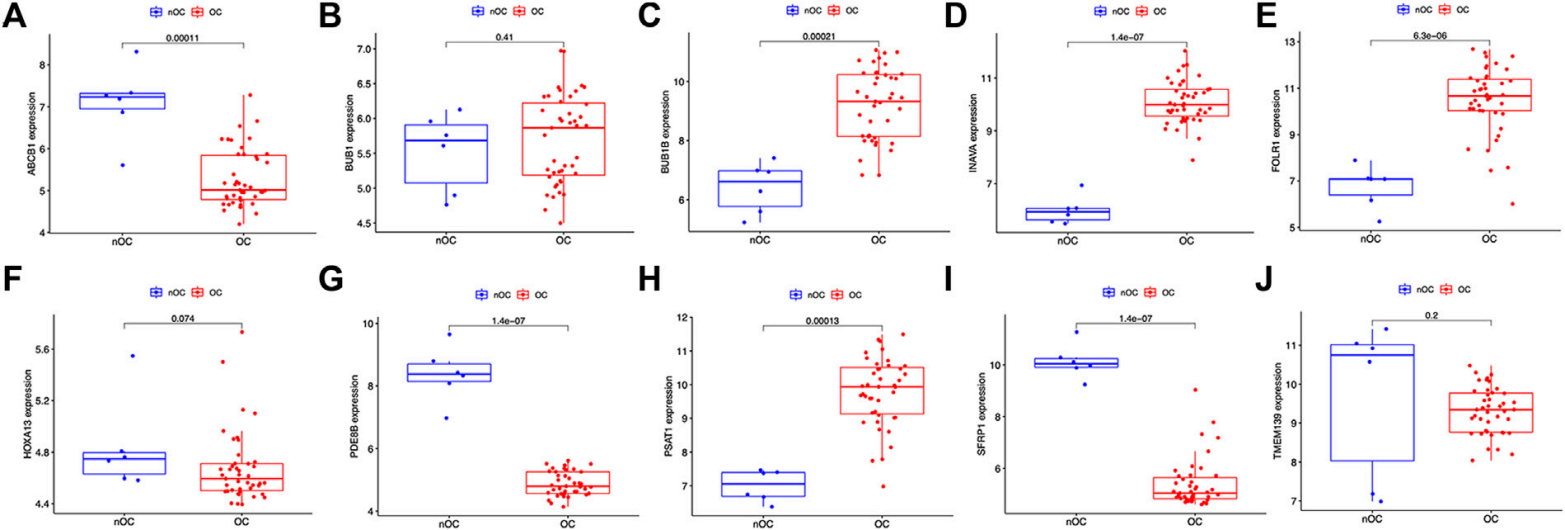
Characteristic genes between the OC group and the nOC group in the training set. **(A-J)** The box plot shows the expression of the intersection genes in the training set between the OC group and the nOC group. Red indicates the ovarian cancer group, and blue indicates the non-ovarian cancer group. *p* < 0.05 indicates differential expression.

**FIGURE 6:**
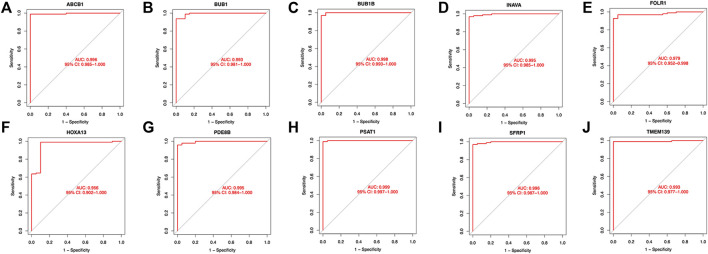
ROC curves of the OC group in the training set. **(A-J)**The figure shows the Roc curve of ten intersection genes in the training set A. The abscissa is the false positive rate, which is represented by 1-specificity, and the ordinate is the true positive rate, which is represented by sensitivity.

Twenty genes were selected by the LASSO algorithm (Figure 4A), 40 genes were selected by the SVM-RFE algorithm (Figure 4B). Finally, their intersection was taken to obtain 10 genes, namely BUB1, ABCB1, SFRP1, INAVA, TMEM139, BUB1B, PSAT1, PDE8B, FOLR1, HOXA13 (Figure 4C). The 10 diagnostic genes obtained were subjected to nOC and OC group difference analysis. OC group is shown in red, nOC group is shown in blue. If *p*-value < 0.05, these potential genes differed between the two groups. In the training set, upregulated potential genes in the OC group were PSAT1, FOLR1, INAVA, BUB1B, and downregulated potential genes were PDE8B, ABCB1, SFRP1 (Figures 5A-J). The diagnostic accuracy of the OC group was verified by ROC curves, and the area under the curve (AUC) of all 10 genes was greater than 0.9 (Figures 6A–J). Besides, there were seven genes (FOLR1, INAVA, BUB1B, ABCB1, SFRP1, PSAT1, PDE8B) with AUC bigger than 0.9. And these seven genes were used as diagnostic genes. (Nuiplot et al., 2015) In validation set A, the AUCs of ABCB1, BUB1B, INAVA, FORL1, PDE8B, PSAT1, and SFRP1 were greater than 0.9 (Supplementary Figure S1). In validation set B, 6 genes were differentially expressed in OC group and nOC group. They were FOLR1, INAVA, BUB1, ABCB1, PSAT1, PDE8B, and TMEM139 (Supplementary Figure S3). The AUCs of all seven genes were greater than 0.9 (Supplementary Figure S4). However, there was no relevant expression of SFRP1 and BUB1B genes detected in the validation set B. Due to the high lethality of ovarian cancer, we took the potential genes obtained from validation set A and validation set B. Thus, altogether, 9 diagnostic genes were obtained.

### Immune Cell Infiltration

**FIGURE 7:**
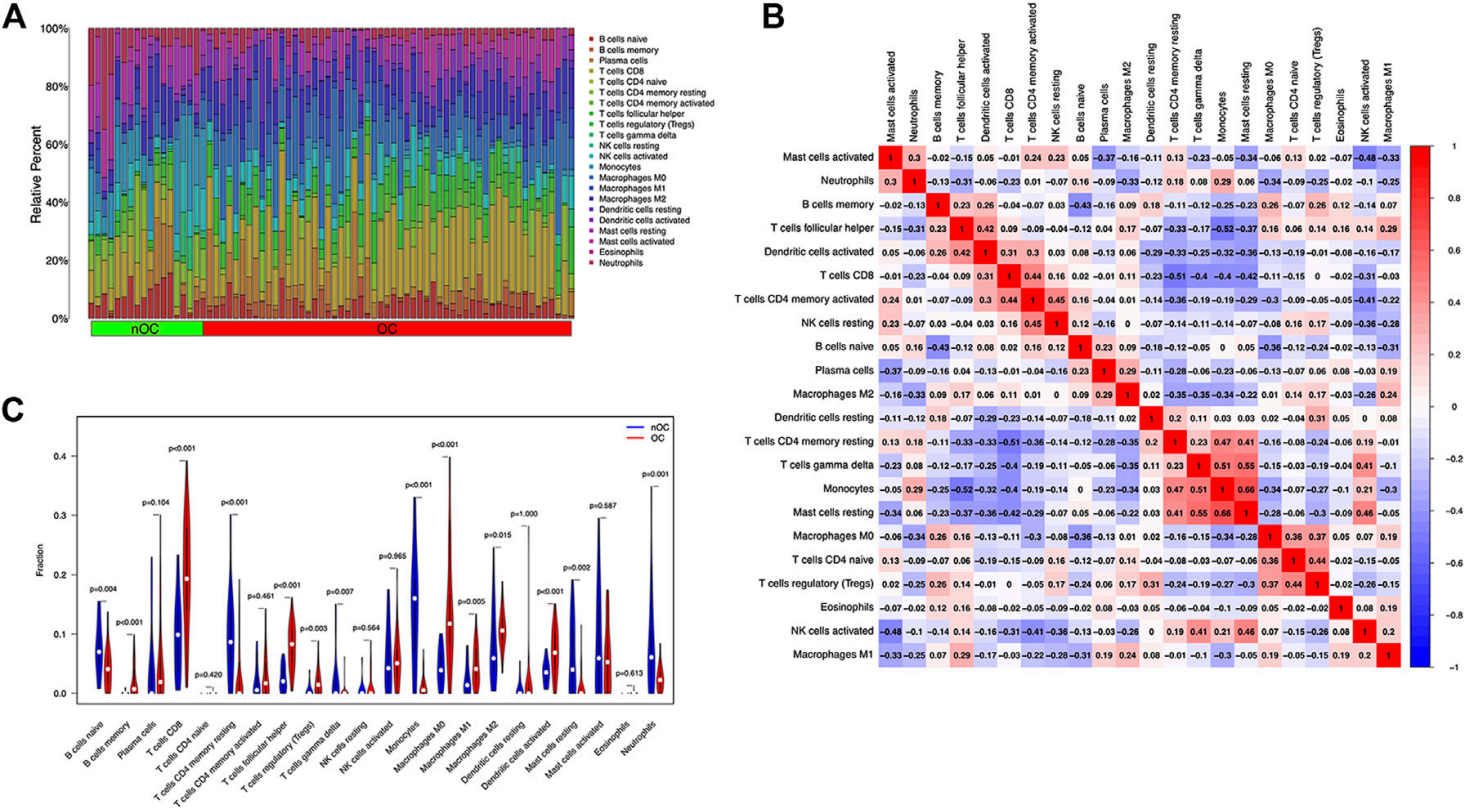
Immune cell infiltration analysis in the training set. **(A)** The graph shows the level of infiltration of different immune cells between the ovarian cancer group and the non-ovarian cancer group. **(B)** The violin chart shows the different analyses of immune cells. The abscissa indicates the name of immune cells, the ordinate indicates the content of immune cells, blue indicates the control group, and red indicates the ovarian cancer group. *p* < 0.05 indicates that there is a significant difference in the content of immune cells between the two groups. **(C)** Correlation analysis between immune cells. Both the abscissa and the ordinate are the names of immune cells, and the value indicates the correlation coefficient between immune cells. Red indicates positive correlation, and blue indicates negative correlation. The two immune cells associated with the red grid, the higher the level of one immune cell, the higher the level of the other immune cell. The higher the level of one of the two immune cells associated with the blue grid, the lower the level of the other immune cell.

**FIGURE 8:**
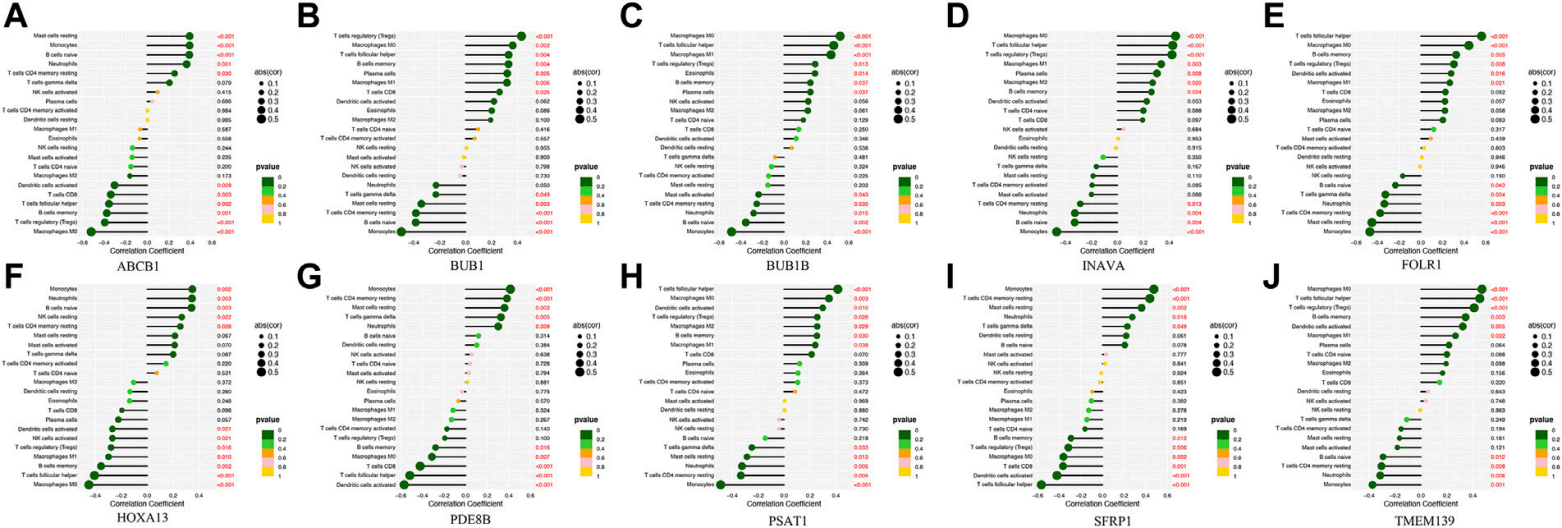
The correlation lollipop plot shows the results of immune cell and target gene correlations. **(A-J)** Association between the diagnostic-related genes and immune cell infiltration. The horizontal coordinate indicates the correlation coefficient, and the vertical coordinate indicates the immune cell name. The circle size indicates the absolute value of the correlation coefficient, the color indicates the *p*-value of the correlation test, and the *p*-value size is indicated by color.

Analysis of the differential expression of immune cells in the OC and nOC groups showed a significant difference in the infiltration of B cells, T cells, macrophage, neutrophils, and non-activated mast cells, *p* < 0.05 (Figure 7A). Immune cell correlation in TME was then visualized in the form of a heat map (Figure 7B). Further analysis of the difference in the degree of cellular infiltration between nOC and OC groups indicated that Memory B cells, CD8^+^ T cell, T follicular helper cells, Regulatory T cells (Tregs), M0 macrophage, M1 macrophage, and dendritic cell activation infiltration were significantly higher in the Oc group than in the nOC group. However, B-cell progenitor, CD4^+^ T cell memory, Gamma delta T cells, monocytes, M2 macrophages, mast cell quiescence, reticulocyte infiltration were significantly lower than that in the nCO group (Figure 7C). After that, we further analyzed the relationship between the screened differential genes and immune cell infiltration (Figure 8). Correlation analysis was performed between genes and immune cells, with the size of the circle representing the absolute value of the correlation coefficient and the color of the circle representing the *p*-value of the correlation test. When *p*-value <0.05, then there is a correlation between immune cells and the target gene (shown in red), which implies that the correlation between the immune cells and the target gene is significant (Figures 8A–J).

### Prognostic Analysis and Protein Expression Analysis

After searching these diagnostic candidate genes on the KM-plotter website, we selected genes with log rank *p* < 0.05 in the survival analysis graph. Finally, we found that BUB1, FOLR1, and PSAT1 among these genes have prognostic effects (Supplementary Figure S5).

We found that five (SFPR1, PSAT1, PDE8B, INAVA, and TMEM139) of the nine diagnostic candidate genes could be retrieved, and their expression trends were similar to our analysis results (Supplementary Figure S6).

## Discussion

Ovarian cancer is one of the most lethal gynecologic malignancies, characterized by high incidence and lethality (Torre et al., 2018). Due to its vague symptoms in the early stage, 70–80% of patients are first diagnosed at a late stage (III-IV) of the disease. Thus the five-year survival rate of patients is significantly reduced (Lheureux et al., 2019). In recent years, with the rapid development of bioinformatics technology, the relationship between genes and tumors has been explored more deeply. There is increasing evidence that alterations in gene expression levels are involved in tumorigenesis and progression. Machine learning is a core discipline of artificial intelligence (AI), which utilizes algorithms that detect patterns within existing data, then train itself to make predictions on new data (Badrick et al., 2019). To identify more useful diagnostic biomarkers in OC group, we used bioinformatics methods to obtain 590 differentially expressed genes between OC and nOC groups. The enrichment analysis of these DEGs indicated they were mainly enriched in a cell cycle as well as proteoglycan-related pathways and cell division-related functions highly associated with gynecologic oncological diseases. Then, nine key genes were screened as candidates for ovarian cancer diagnosis by two machine learning algorithms, LASSO and SVM. Further immunoassays revealed significant differences in the infiltration of B cells, T cells, macrophages, neutrophils, and non-activated mast cells between OC and nOC groups. These results provide new insights into the diagnosis and treatment of ovarian cancer.

The 590 DEGs were used for GO, KEGG, GSEA, and DO analyses. KEGG pathway analysis indicated that these DEGs were mainly involved in the cell cycle, proteoglycan, progesterone-mediated oocyte maturation, fluid shear stress, atherosclerosis, cell adhesion molecules, and IL-17 pathways. These pathways are closely associated with ovarian carcinogenesis and progression. Abnormalities in cell cycle mechanisms often accompany ovarian carcinogenesis. In the early stages of ovarian cancer, the process of cell nuclear division with diminished ability to excise damaged bases, as well as homologous recombination and diminished chromosome repair, promotes cells to enter the S phase from G0/G1 phase. It accelerates tumor cell proliferation and growth (Liu et al., 2019). Interestingly, we obtained similar results in GSEA and GO analyses. GSEA analysis revealed that the top five active pathways in OC were base excision repair, cell cycle, cysteine and methionine metabolism, DNA replication, and homologous recombination pathways, mainly concentrated in the stage of ovarian carcinogenesis and cell metabolism-related pathways. GO enrichment analysis also revealed that these DEGs were primarily associated with cell nuclear division, cell cycle, extracellular matrix, and epithelial growth. In addition to cell cycle abnormalities, the metastatic mechanism of OC is equally extremely important. Unlike most advanced tumor types that metastasize through blood vessels, it metastasizes mainly through the intra-abdominal cavity by the luminal route (Hassan et al., 2020). In the peritoneal cavity, tumor-mesothelial adhesions, as well as cell-to-cell interactions, are important steps in the spread of the tumor. Selectins as a glycan-binding molecule play an essential role in ovarian cancer intraperitoneal metastasis, as do cell adhesion molecules. Fluid dynamics mediates heterotypic cell-cell interactions underflow, facilitating the early steps of this adhesion cascade. Besides, related studies have shown that ovarian is a critical endogenous factor in inducing the progression of primary tumors to metastatic ovarian cancer. Blocking progesterone signaling by the pharmacological inhibitor, mifepristone, inhibits the development of HGSC (high-grade plasmacytoma) and its metastasis to the peritoneum as well as to the ovary effectively (Kim et al., 2020). The literature suggests that OC patients have a higher survival rate if they have IL-17-secreting (Th17s) T cells in their bodies (Block et al., 2020). DO analysis revealed that DEGs PSAT1, FOLR1, ABCB1, SFRP1, SFRP1, BUB1B have a greater association with female reproductive system tumor-related diseases, such as malignant ovarian surface epithelial-mesenchymal tumor, ovarian epithelial carcinoma, and ovarian cancer. SFRP1 and FOLR1 have a greater association with urologic tumors, like renal cell carcinoma or kidney cancer.

After further screening by two machine learning algorithms, a total of nine genes with diagnostic values were selected: SFRP1, PSAT1, BUB1B, FOLR1, ABCB1, PDE8B, INAVA, BUB1, and TMEM139. Secreted frizzled-related protein 1 (SFRP1) plays an important role in tumorigenesis, acting as a negative regulator of Wnt signaling (Cheng et al., 2017). It was found that the SFRP1 gene, as a potential tumor suppressor, was down-regulated by epigenetic alteration of SFRP1 protein expression. Besides, abnormal activation of the SFRP1/Wnt pathway leads to ovarian tumorigenesis. On the contrary, inhibiting the Wnt/β-catenin pathway suppresses epithelial ovarian cancer. Phosphoserine aminotransferase (PSAT1) is a serine catalase that plays an important role in the development of ovarian cancer. Serine-related proteins are overexpressed in epithelial ovarian cancer (EOC) compared to normal ovarian tissue. Their expression correlates with tissue subtype, FIGO stage, histological grade, lymph node metastasis, distant metastasis, and metastasis in the presence of ascites (Zhang et al., 2020). *In vitro* experiments, downregulation of PSAT1 inhibited the growth of EOC cells and induced apoptosis and cell cycle arrest (Dai et al., 2019). Recently, increasing evidence demonstrated that PSAT1 was overexpressed in OC with poor prognosis (Zheng et al., 2019). Mitotic checkpoint serine/threonine kinase B (BUB1B) is a conserved multifunctional protein essential for mitotic spindle checkpoint and correction of kinetic-microtubule junctions. BUB1B variants cause ovarian insufficiency and early menopause. Several studies have shown that highly elevated BUB1B is associated with high OC cell proliferation and poor clinical prognosis (Feng et al., 2019; Chen et al., 2020). Folate receptor 1 (FOLR1) is a glycosylphosphatidylinositol (GPI)-anchored glycoprotein, enriched in oocytes of primary, secondary, and tertiary follicles as well as in surrounding granulosa cells. FOLR1 is expressed in rapidly growing solid malignancies as well as in most ovarian cancers (Köbel et al., 2014). Related experiments have shown that single-dose bispecific targeting of FOLR1 and death receptor 5 (DR5) is an effective strategy for treating ovarian cancer, suggesting FOLR1 may be a potential biomarker for ovarian cancer (Shivange et al., 2018). ATP Binding Cassette Subfamily B Member 1 (ABCB1) encodes a multidrug resistance protein (MDR1). It is involved in the cellular exocytosis of chemotherapeutic drugs, and it may be used as an alternative marker for the diagnosis of ovarian cancer progression. Moreover, it also correlates with ovarian cancer resistance, healing, and prognosis (Zhou et al., 2019). Many drug-resistant recurrent ovarian cancers are associated with the upregulation of ABCB1. Targeted regulation of ABCB1 expression can sensitize ovarian cancer cells to paclitaxel and cisplatin, providing an effective treatment option for patients with chemoresistant ovarian cancer (Sun et al., 2015; Vaidyanathan et al., 2016). The phosphodiesterase (PDE) family is a group of enzymes that catalyze the conversion of cyclic nucleotides to 5′ nucleotides (Petersen et al., 2015). PDE8 is one of the major PED in human ovaries, and has a significantly altered lipogenic and cholesterolemia gene expression profile in ovarian cancer cells. Downregulation of the steroidogenic regulator PDE8B, for example, explains the increased membrane fluidity in ovarian cancer cells, which has potential applications in the development of new biomarkers and treatment of ovarian cancer and deserves further investigation (Pampalakis et al., 2015). INAVA, known as Innate Immunity Activator, is a risk gene for inflammatory bowel disease specifically expressed by mucosal surface epithelial cells. Recent studies have shown that it is associated with tumorigenesis and inflammatory responses (Chang et al., 2021). INAVA expression is associated with regulating two transcription factors, ELF5 and GATA3, which are important in breast stem cells, and targeting INAVA has therapeutic value in breast cancer (Ma et al., 2019). Furthermore, in inflammatory bowel disease, INAVA was used for mitogen-activated protein kinase (MAPK), nuclear factor kappa-B (NF-κB) activation, cytokine secretion, and intracellular bacterial clearance after pattern recognition receptor (PRR) stimulation (Yan et al., 2017). The protein, Threonine-protein kinase (BUB1), was bound to kinetochores and played a key role in establishing the mitotic spindle checkpoint and chromosome congression. In ovarian cancer, BUB1 showed high-level expression (Feng et al., 2019). Down-regulation of BUB1 expression levels suppressed ovarian cancer progression (Jin & Ye, 2021). Transmembrane Protein 139 (TMEM139) has no relevant studies in ovarian cancer. However, two available articles were related to human kidney isoform of anion exchanger 1 (kAE1) (Nuiplot et al., 2015) and papillary thyroid carcinoma. In papillary thyroid carcinoma, TMEM139 was a potential independent predictive gene for the recurrence of PTC (He et al., 2020).

The tumor microenvironment is a complex ecological environment in which the interactions between tumor cells, immune cells, and non-immune cells determine the tumor progression (Hornburg et al., 2021). Among them, immune cells play an important role and greatly influence the invasive and metastatic ability of tumors (Giraldo et al., 2019; van Vloten et al., 2022). Although an increasing number of studies have focused on analyzing the prognostic features of tumors. Yan et al. (2020) constructed a prognostic feature model of ovarian cancer based on immune cell infiltration (Yan et al., 2020). Zhao et al. (2022) constructed a prognostic model of ovarian cancer through WGCNA and machine learning methods (Zhao et al., 2022). However, there is no research to analyze the diagnostic signature genes of ovarian cancer through machine learning algorithms and medical big data. In this study, a machine learning algorithm was used to screen out ovarian cancer genes with diagnostic characteristics, and the correlation between these genes and immune cells was also analyzed. These results help to improve the diagnostic specificity of ovarian cancer patients, and at the same time can inform us which genes may influence the immunotherapy of ovarian cancer patients. In this study, we found that the highest level of infiltration in the tumor microenvironment of ovarian cancer patients is CD8^+^ T lymphocytes. CD8^+^ T lymphocytes are typical anti-tumor immune cells that specifically label cytotoxic T cells that play an active role in tumor clearance while recognizing and secreting cytotoxic molecules to kill tumor cells. This process of cytotoxicity by CD8^+^ T cells needs to be initiated by dendritic cells. In breast cancer studies, investigators found that high infiltration of CD8^+^ T cells was associated with more prolonged survival. Tumor-associated macrophages (TAMs) are macrophages in tumor tissues. Different types of TAMs have different characteristics. M1 TAMs are characterized by high expression of IL-12 and low expression of IL-10, which can present tumor-specific antigens and inhibit tumor growth (Mantovani et al., 2017). In contrast, M2 TAMs have high expression of IL-10 and low expression of IL-12, which can promote tumor growth and are resistant to chemotherapy (Lee et al., 2019). Different types of macrophages in ovarian cancer have different effects on their prognosis, and studies have shown that high density of CD163 + M2 TAMs is associated with advanced stage and poor prognosis in epithelial ovarian cancer (Reinartz et al., 2014). This study also found more M2 macrophages in ovarian cancer patients than in non-ovarian cancer patients. M2 macrophages can promote tumor invasion and metastasis by inducing stromal cell proliferation, angiogenesis, and extracellular matrix deposition (Qian et al., 2011; Pyonteck et al., 2013). In addition, some studies have found that macrophages and B cells are associated with prolonged survival in patients with non-small cell lung cancer (Ohri et al., 2009; Lohr et al., 2013; Hernández-Prieto et al., 2015). The composition of the tumor microenvironment is complex and variable, and we cannot only explore the tumor-promoting or tumor-suppressing effects of one type of immune cells, but further studies are needed to explore the interactions among immune cells and between immune cells and other cells.

Despite the use of bioinformatics and machine learning algorithms in our study and the discovery of the diagnostic value of key genes in ovarian cancer patients, there are still some limitations. First of all, the data in this study comes from the GEO database, which requires more data from different databases to verify. To reduce the deviation of a single data set, we used three GEO data sets for this study. Secondly, since this study only utilizes data from online databases, the results may be biased. We need to collect complete data for research and further experiments to verify. Finally, our study was only explored at the genetic level, and multi-omics data or immune-related non-coding RNA signatures may help us gain insight into the pathogenesis of ovarian cancer and better predict survival.

## Conclusion

In summary, we identified 10 characteristic genes of ovarian cancer using bioinformatics methods and two machine learning algorithms, and also explored the biological functions and pathways involved in these genes. Nine key characteristic genes were then screened by metrics such as AUC. Notably, the characteristic genes validated in our study may be associated with different levels of immune infiltration in ovarian cancer patients. A better understanding of the immune status of cancer is essential to advance treatment progress. As clinical trials of immunotherapeutic agents and their various combinations progress, this approach may provide some reference value to develop reliable guidelines for drug selection, increase treatment response, and help clinicians manage patients with ovarian cancer.

## Data Availability

Publicly available datasets were analyzed in this study. This data can be found here: https://www.ncbi.nlm.nih.gov/geo/. GSE12470, GSE18520, and GSE27651. Also found in TCGA database (http://portal.gdc.cancer.gov/) and GETx database (www.gtexportal.org).

## Supplementary Material

Supplementary Figure S1 The figure shows the Roc curve of 10 diagnostic genes in the validation set A.

Supplementary Figure S2 10 Diagnostic genes and immune cell infiltration correlation test.

Supplementary Figure S3 Characteristic genes between the OC group and the nOC group in the validation set B.

Supplementary Figure S4 ROC curves of the OC group in the validation set B.

Supplementary Figure S5 Diagnostic candidate genes on the KM-plotter.

Supplementary Figure S6 Protein expression analysis.

Supplementary Table S1 Briefly describe of each data set.
