# Supplementary figures and images for: Identification and Validation of the Diagnostic Characteristic Genes of Ovarian Cancer by Bioinformatics and Machine Learning
Source: Front Genet. 2022 Jun 1;13:858466. doi: 10.3389/fgene.2022.858466 (PMC9198487; doi:10.3389/fgene.2022.858466)

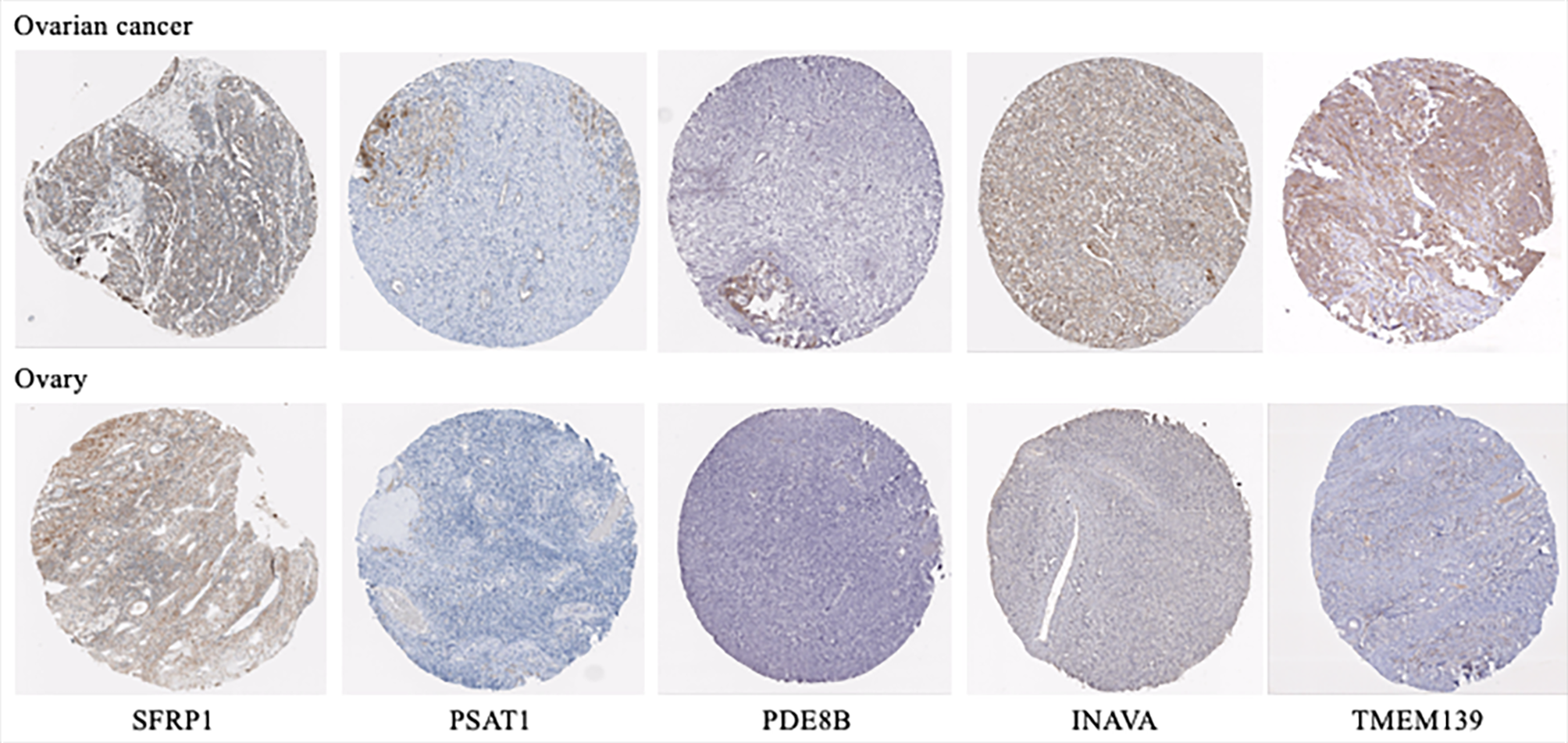

Supplement: Supplementary file 1 [file Image6.TIF]

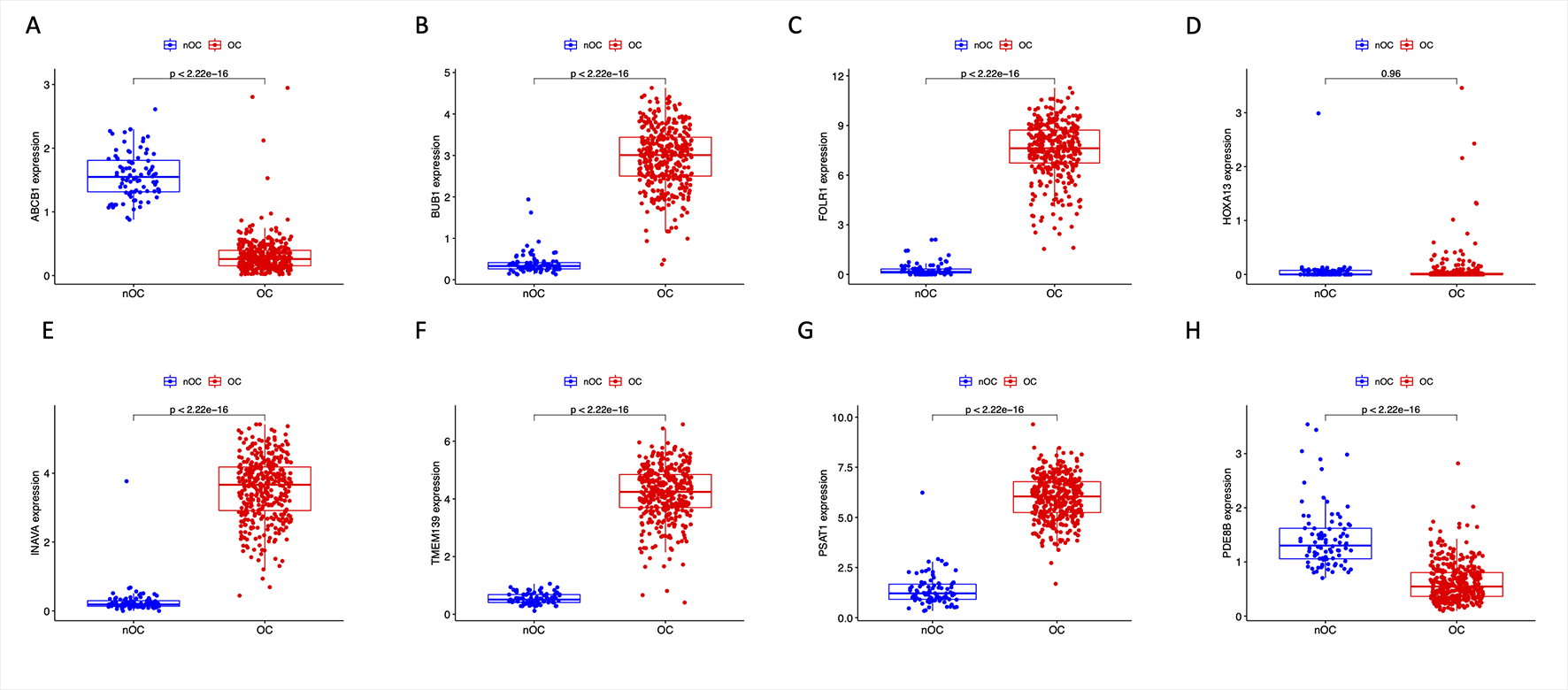

Supplement: Supplementary file 3 [file Image3.TIF]

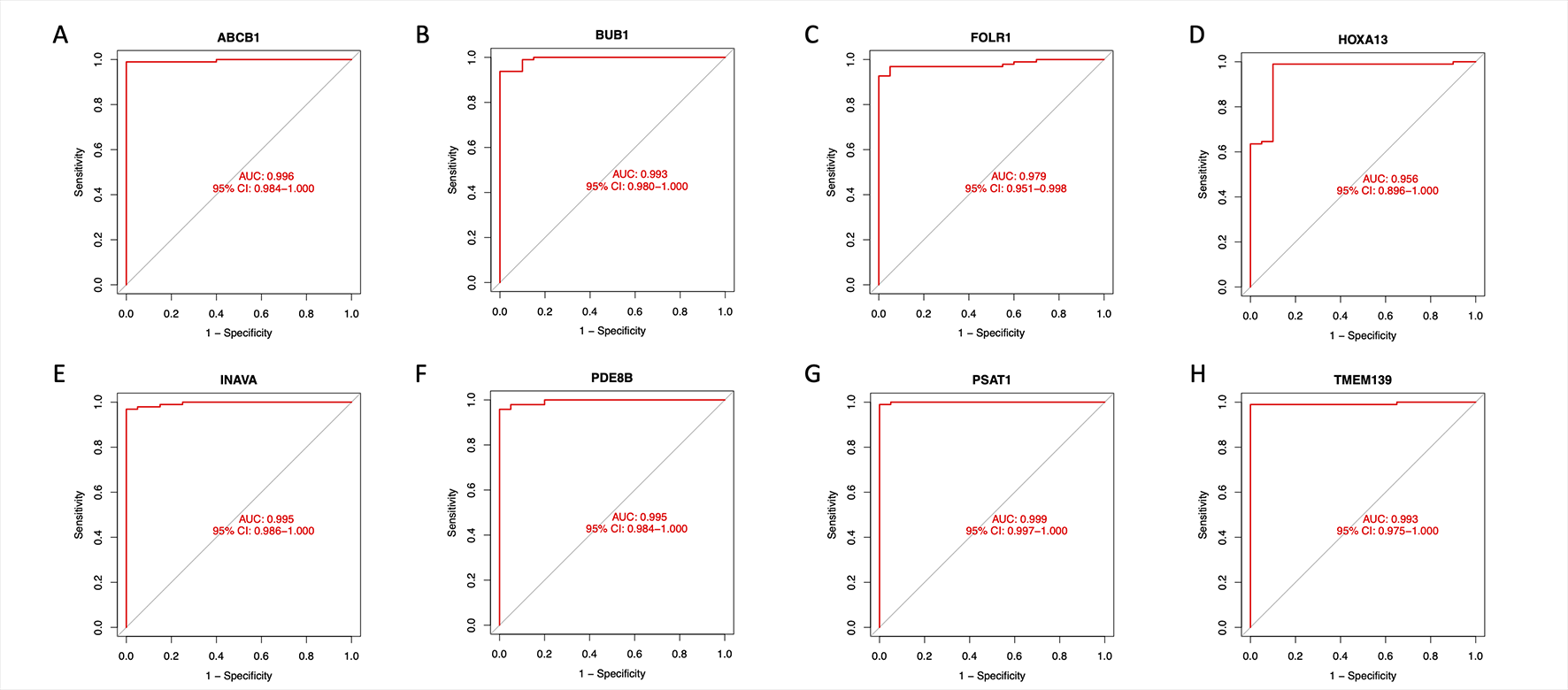

Supplement: Supplementary file 4 [file Image4.TIF]

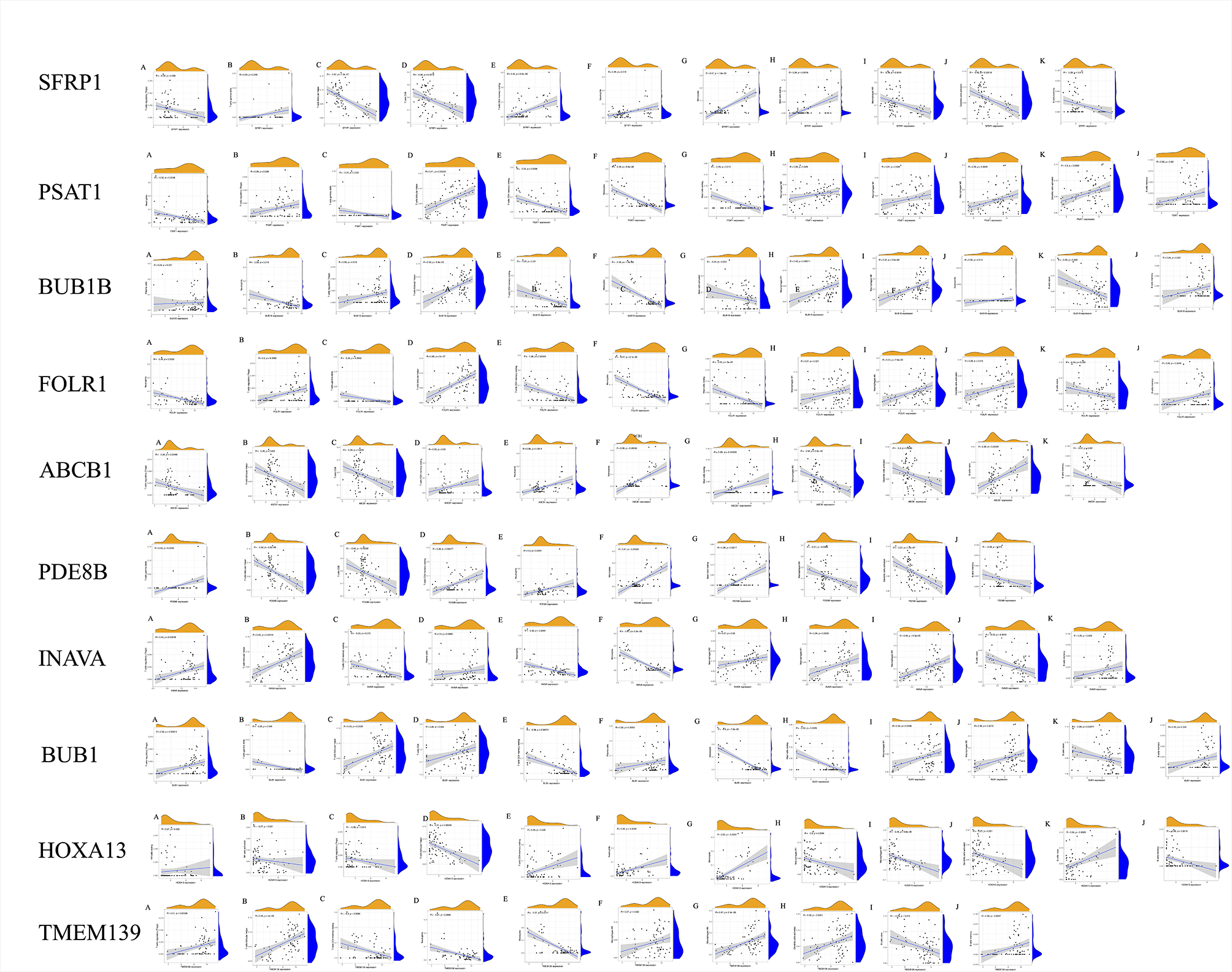

Supplement: Supplementary file 6 [file Image2.TIF]

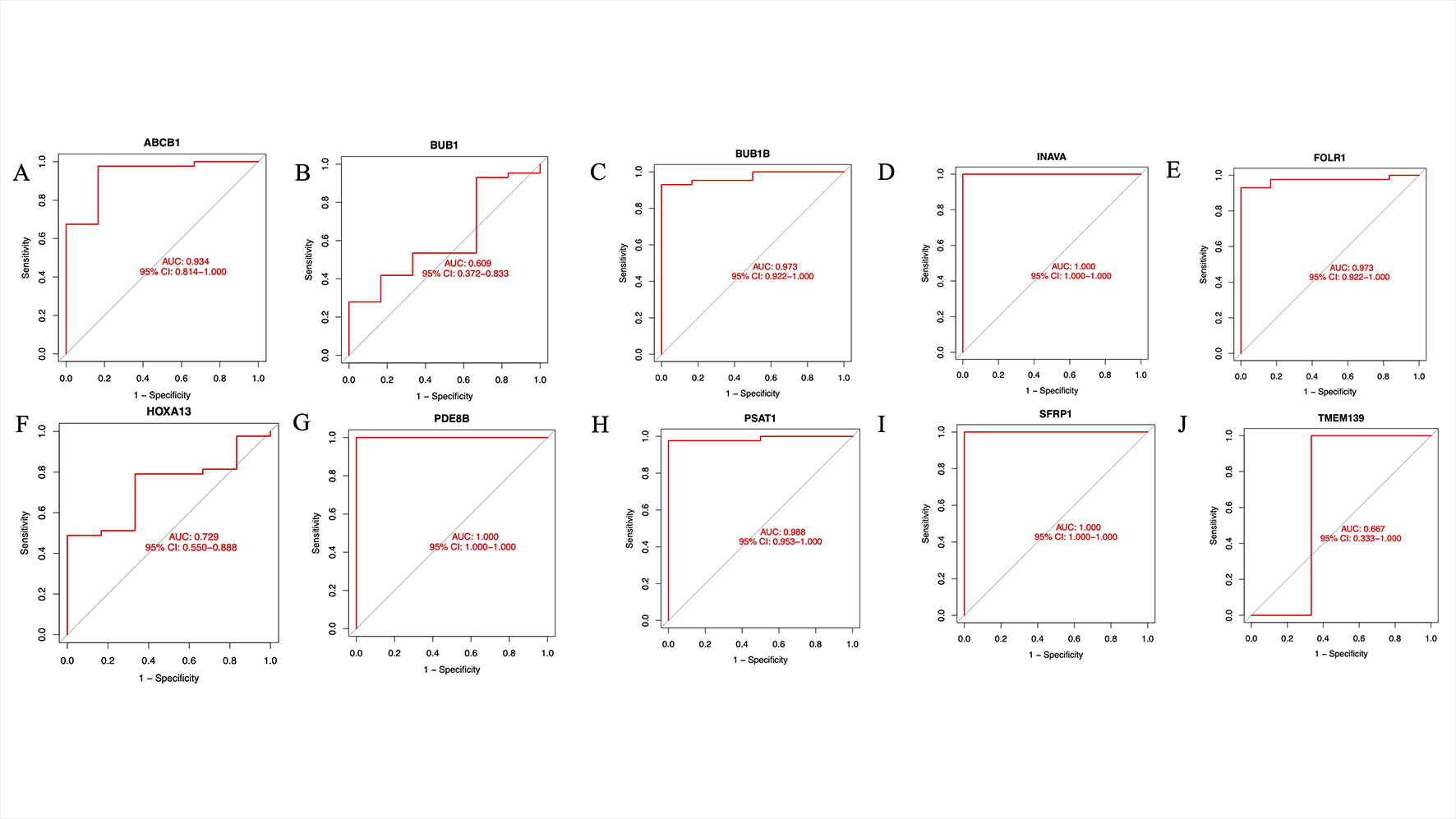

Supplement: Supplementary file 7 [file Image1.TIF]

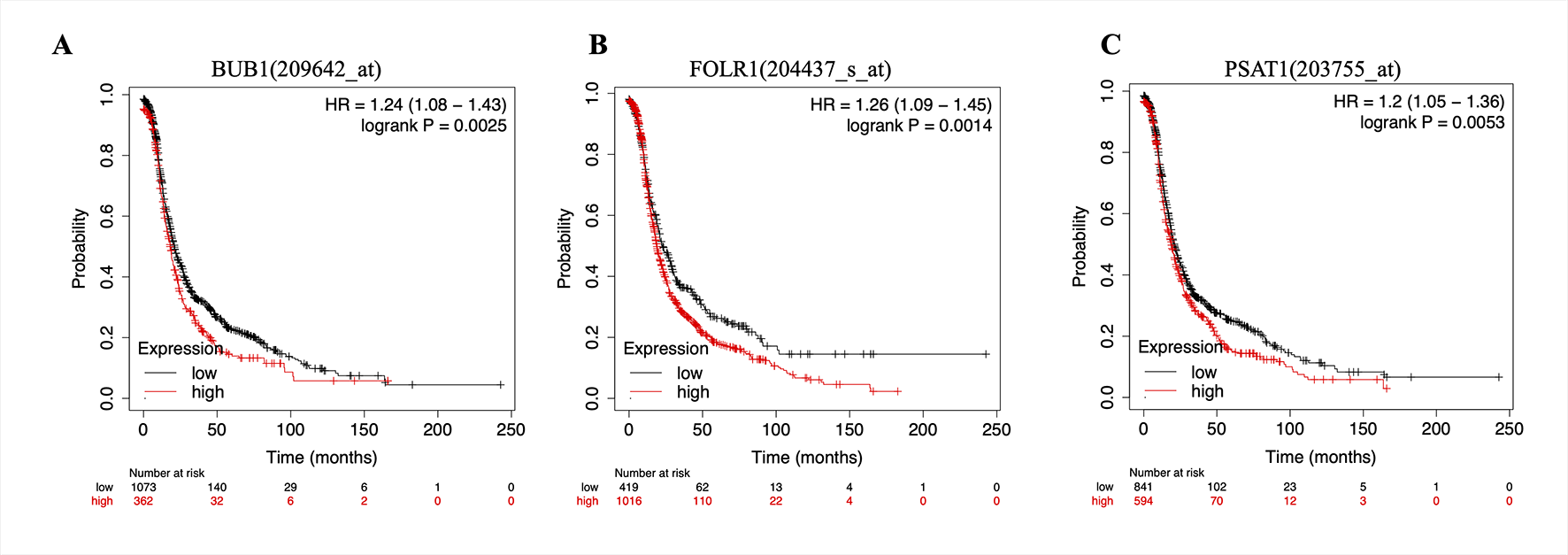

Supplement: Supplementary file 8 [file Image5.TIF]
