# Supplementary material for: Identification and Validation of the Diagnostic Characteristic Genes of Ovarian Cancer by Bioinformatics and Machine Learning
Source: Front Genet. 2022 Jun 1;13:858466. doi: 10.3389/fgene.2022.858466 (PMC9198487; doi:10.3389/fgene.2022.858466)
Supplement: Supplementary file 2 [file Table1.DOCX]

|  | Number of samples Stage | | | | | | |  | Grade | |  |  |  |  |  |  |
| --- | --- | --- | --- | --- | --- | --- | --- | --- | --- | --- | --- | --- | --- | --- | --- | --- |
| Data set ID | Country | | Tumor | Normal | | I | II-IV | G1 | G2 | G3 G4 unknown | |  |  |  |  |  |
| GSE12470  GSE18520  GSE27651  TCGA  GTEx | | Japan  USA  USA | 43  53  43 | 10  10  6  379  88 | 8  0  0 | | 35  53  49 | 1 | 44 323 1 | | 10 |  |  |  |  |  |
